# Validation of a Microsphere Immunoassay for Serological Leptospirosis Diagnosis in Human Serum by Comparison to the Current Gold Standard

**DOI:** 10.1371/journal.pntd.0003636

**Published:** 2015-03-25

**Authors:** Sarah J. Wynwood, Mary-Anne A. Burns, Glenn C. Graham, Steven L. Weier, David B. McKay, Scott B. Craig

**Affiliations:** 1 Faculty of Science, Health and Education, University of the Sunshine Coast, Sippy Downs, Queensland, Australia; 2 WHO/OIE/FAO Collaborating Centre for Reference and Research on Leptospirosis, Queensland Health Forensic and Scientific Services, Archerfield, Queensland, Australia; 3 Chemical Analysis Unit, Queensland Health Forensic and Scientific Services, Archerfield, Queensland, Australia; 4 School of Biomedical Sciences, Queensland University of Technology, Brisbane, Australia; Small, University of Tennessee, UNITED STATES

## Abstract

A microsphere immunoassay (MIA) utilising Luminex xMap technology that is capable of determining leptospirosis IgG and IgM independently was developed. The MIA was validated using 200 human samples submitted for routine leptospirosis serology testing. The traditional microscopic agglutination (MAT) method (now 100 years old) suffers from a significant range of technical problems including a dependence on antisera which is difficult to source and produce, false positive reactions due to auto-agglutination and an inability to differentiate between IgG and IgM antibodies. A comparative validation method of the MIA against the MAT was performed and used to determine the ability of the MIA to detect leptospiral antibodies when compared with the MAT. The assay was able to determine samples in the reactive, equivocal and non-reactive ranges when compared to the MAT and was able to differentiate leptospiral IgG antibodies from leptospiral IgM antibodies. The MIA is more sensitive than the MAT and in true infections was able to detect low levels of antibody in the later stages of the acute phase as well as detect higher levels of IgM antibody earlier in the immune phase of the infection. The relatively low cost, high throughput platform and significantly reduced dependency on large volumes of rabbit antisera make this assay worthy of consideration for any microbiological assay that currently uses agglutination assays.

## Author Summary

Leptospirosis is a zoonotic disease caused by spirochaetes of the genus *Leptospira* and affects millions of people, worldwide, each year. Laboratory diagnosis of leptospirosis currently relies on methods that are flawed in many areas. Current methods are outdated, time consuming and expensive. They rely on a continuous supply of animal products (rabbit anti-sera) and require specialist expertise and equipment. The current gold standard diagnostic assay for leptospirosis (MAT) cannot determine IgG from IgM antibodies and relies on live cultures, which presents problems in the way of maintenance and attenuation. Development of a new diagnostic assay for serological diagnosis of leptospirosis that is specific, sensitive and able to discriminate between IgG and IgM classes of antibodies—as well as being more cost effective—will significantly improve the capabilities for detecting leptospirosis infections. It will provide medical professionals with more valuable diagnostic information and public health professionals with improved epidemiological information.

## Introduction

Leptospirosis is considered to be the most widespread zoonotic disease in the world [1] with clinical diagnosis proving challenging due to the non-specific nature of symptoms associated with the disease. There are some 300 leptospiral serovars belonging to a number of different serogroups. Currently there are 24 sero-groups of pathogenic leptospires based on their antigenic relatedness [2]. Leptospirosis was first reported in Australia in 1933 in the state of Queensland and has since been isolated Australia wide [3] with Queensland reporting the majority of these cases (57.6%) [4]. In 2011 the reported incidence of leptospirosis in Queensland was 3.4 cases per 100,000 people and overall in Australia the incidence was 0.84 cases per 100,000 people [5]. At present, 24 serovars of *Leptospira spp* are recognised in Australia and in recent years a dramatic increase in the incidence of leptospirosis cases in Australia (particularly Queensland) has been noted with environmental factors believed to be the main influence on this increase [6].

Diagnosis of leptospirosis occurs at two stages—during the acute phase the live organism can be detected by two methods. Polymerase chain reaction (PCR) testing is a useful molecular detection tool for rapid qualitative diagnosis of leptospirosis in its earliest stage [7]. Serum or blood samples provided for PCR testing must be collected within a precise timeframe (0–8 days post onset) to enable diagnosis. Blood culture isolation can also be utilised in the early stages of leptospiral infection (0–10 days post onset), however this method is time consuming, requires specialised media and equipment and can take months for a serovar specific result [8]. The immune phase of a leptospiral infection is characterised by the presence of leptospiral antibodies and diagnosis is based on serological methods with the microscopic agglutination test (MAT) considered the current gold standard [9]. If the stage of the disease is unknown, both acute and immune phase tests are performed. Other serological test methods have previously been developed including flow cytometry [10], complement fixation testing [11], indirect hemagglutination assay [12] an IgM dipstick assay [13] and an IgM enzyme-linked immunosorbent assay (ELISA) in a number of formats [14,15]. Each of these assays has its advantages and disadvantages [16] and the type of assay used for diagnosis is generally dependant on the facilities available.

Serological diagnosis of leptospirosis in humans in Queensland, Australia is currently performed by screening with a commercially available leptospirosis IgM ELISA followed by the MAT as a reference and confirmatory test. The MAT method has many disadvantages as it requires specialist expertise, fresh leptospirosis cultures, is labour intensive, costly and is capable of determining total antibody only. The current endemic routine panel for MAT testing in Queensland, Australia consists of 16 serovars, with representatives from a number of different serogroups. Each sample submitted for MAT is screened against this panel and any reactive samples are then serially diluted and retested to determine an end point. Results are reported as a titre with the end point being the final dilution of serum at which 50% or more of the leptospires are agglutinated. This assay permits the testing of up to 20 samples per day on a routine basis. The MIA has the ability to simultaneously test large numbers of samples against large numbers of serovars as well as determine individual IgG and IgM titres. These factors alone would be enormously beneficial in the laboratory diagnostics and epidemiological studies of leptospirosis.

Bead based suspension array technology (xMap, Luminex) has the capacity to multiplex up to 500 individual analytes in a single well and has been shown to be a successful diagnostic tool for serology in many applications [17,18,19]. This assay platform is based on magnetic coated polystyrene beads filled with two coloured fluorescent dyes in differing ratios resulting in 500 distinct bead sets. Each bead set can be coated with a different antigen and mixed to allow the simultaneous measurement of antibody response to up to 500 different antigens. This high-throughput screening system allows processing of high numbers of patient samples per day. Its speed, sensitivity, and accuracy of multiple binding events measured in the same small volume have the potential to replace many clinical diagnostic and research methods and deliver data on hundreds of analytes simultaneously [20]. The microsphere immunoassay (MIA) that has been validated in this study was adapted from the method described by Luminex Corp (2000) and can be utilised as a routine serology testing protocol for leptospirosis.

The development and validation of a high quality, reliable serological assay is pertinent to the ability of a laboratory to sero-diagnose diseases in humans. Assay development begins with the identification of a need for improved diagnostic capabilities and the benefits that can be obtained from such an assay. A Luminex microsphere immunoassay (MIA) for leptospirosis antibody detection has the potential to function both as a high sensitivity, high throughput screening assay as well as a high specificity assay for determination of serovar level antibodies. This paper assesses the leptospirosis MIA in human samples as a screening assay to determine reactive, equivocal and non-reactive samples. Validation is performed by comparison to the leptospirosis IgM ELISA and the current gold standard, the microscopic agglutination test (MAT) as the basis for defining the performance characteristics of the MIA.

## Materials and Methods

### Ethics

The study protocol was approved by the Public and Environmental Health Research Committee and the Humans Ethics Committee, Queensland Health Forensic and Scientific Services. All human samples utilised in this study were de-identified and allocated a generic number.

### Antigens

Sixteen Australian endemic pure leptospiral cultures, Table 1, were grown for 5–7 days in 3mL EMJH broth at 30^°^C. These antigens were then quantitated using a Petroff-Hausser grid and centrifuged at 4^°^C for 25 mins. The supernatant was removed and the pellet resuspended in 500μL phosphate buffered saline (pH 7.5). All cultures were then diluted to obtain a concentration of 1.8 x 10^9^ per mL. These diluted antigens were used to coat 16 individual Bio-Plex Pro Magnetic COOH Bead-sets. Coupled beads were then checked for sensitivity and specificity using rabbit anti-sera of known serovar and titre, obtained from MAT results (See method below).

**Table 1:** Leptospiral cultures (antigens) used for assay validation.

*L*. *Interrogans* serovar Pomona	*L*. *Kirschneri* serovar Grippotyphosa
*L*. *Borgpetersenii* serovar Hardjobovis	*L*. *Weilii* serovar Celledoni
*L*. *Borgpetersenii* serovar Tarassovi	*L*. *Interrogans* serovar Szwajizak
*L*. *Interrogans* serovar Australis	*L*. *Interrogans* serovar Medanensis
*L*. *Interrogans* serovar Zanoni	*L*. *Kirschneri* serovar Bulgarica
*L*. *Interrogans* serovar Robinsoni	*L*. *Interrogans* serovar Copenhageni
*L*. *Interrogans* serovar Canicola	*L*. *Borgpetersenii* serovar Arborea
*L*. *Interrogans* serovar Kremastos	*L*. *Weilii* serovar Topaz

### Serum Samples

This study utilised 200 serum samples which were selected from human serum samples submitted for routine leptospirosis serology to the WHO/FAO/OIE Collaborating Centre for Leptospirosis Reference and Research during 2012 and 2013. These samples were submitted from Queensland hospitals and private laboratories. One hundred and eighty of these samples had leptospirosis IgM ELISA reactive serology, 12 had non-reactive leptospirosis IgM ELISA serology and the remaining 8 samples were not tested previously using leptospirosis IgM ELISA. All leptospirosis IgM ELISA testing was performed at a Queensland hospital or private laboratory prior to the samples being received at the WHO/FAO/OIE Collaborating Centre for Leptospirosis Reference and Research. Routine diagnostic MAT was performed on all samples at the WHO/FAO/OIE Collaborating Centre for Leptospirosis Reference and Research and results recorded against 16 routinely used, endemic serovars.

Forty-eight additional samples with reactive serology for Dengue Virus (24), Barmah Forest Virus (8), Ross River Virus (8) or Rabies Virus (8) antibodies were obtained from the Queensland Health Public and Environmental Health Virology Laboratory. These samples had previously been tested by ELISA IgM (Dengue virus), ELISA IgG (Rabies virus) or Alphavirus Hemagglutination Inhibition total antibody (HAI) (Ross River virus and Barmah Forest virus) and were used to assess whether cross reactions exist in the leptospirosis MIA.

In addition to the 200 samples used for the validation, 20 sets of paired samples with a non-reactive leptospirosis acute sample and reactive leptospirosis convalescent sample on the MAT were also obtained and analysed using the MIA to determine a timeline for the detection of leptospiral antibody. The results for these twenty additional samples are shown separately.

### Bead Coupling

Leptospiral antigens were covalently coupled to individual Bio-Plex Pro Magnetic COOH bead-sets (Table 2) using the Bio-Rad Amine Coupling kit and methods from Luminex Corp. Coupling is achieved via carbodiimide reactions involving the primary amino groups on the protein and the carboxyl functional groups on the bead surface. The bead yield per coupling reaction is approximately 2,500 beads per well (in a 96-well microtitre plate). For optimum results in the MIA, the coupled beads were diluted 1:4 in Triton-X detergent and 100 beads in 100μL buffer were used for the immunoassay. Each individual coupled bead-set was diluted in phosphate buffered saline (PBS) to give a reading of approximately 100 beads per bead-set per well. The working dilution and specificity of each bead-set was validated prior to use in a diagnostic capacity by utilising serovar-specific rabbit antisera and the IgG method as described below, substituting the secondary antibody with an anti-rabbit IgG (RPE). Bead-sets were considered to be valid for use if the targeted serovar produced an antibody response to that specific bead-set.

**Table 2:** Antigens and their corresponding bead-sets.

Antigen (Culture)	Bio-Plex Bead-set #
*L*. *Interrogans* serovar Pomona	45
*L*. *Borgpetersenii* serovar Hardjobovis	27
*L*. *Borgpetersenii* serovar Tarassovi	35
*L*. *Kirschneri* serovar Grippotyphosa	54
*L*. *Weilii* serovar Celledoni	55
*L*. *Interrogans* serovar Copenhageni	46
*L*. *Interrogans* serovar Australis	26
*L*. *Interrogans* serovar Zanoni	28
*L*. *Interrogans* serovar Robinsoni	34
*L*. *Interrogans* serovar Canicola	52
*L*. *Interrogans* serovar Kremastos	36
*L*. *Interrogans* serovar Szwajizak	44
*L*. *Interrogans* serovar Medanensis	43
*L*. *Kirschneri* serovar Bulgarica	53
*L*. *Borgpetersenii* serovar Arborea	20
*L*. *Weilii* serovar Topaz	29

### Microsphere Immunoassay

Two microsphere immunoassays (IgG and IgM) were performed on 200 serum samples taken from the routine MAT submissions which included samples with MAT titres (serial dilutions) ranging from < 1:50 (non-reactive) to 1:6400. Samples with an MAT titre between 1:50 and 1:200 were considered equivocal and samples with a titre 1:400 or above were considered reactive. Pooled convalescent serum from patients with recent leptospirosis infections, confirmed by PCR (on acute sample) and MAT, was used as the positive control serum in each microsphere immunoassay. Negative patient serum, confirmed by negative PCR and serology) was pooled and used as negative control serum. These controls were monitored each run to ensure the assay was consistent.

A 96-well filter plate was pre-wetted with 150μL PBS per well and vacuum applied. One-hundred μL of the diluted coupled beads were then added to each required well of the pre-wetted 96-well microtitre filter plate and vacuum applied. Serum samples for the IgG immunoassay were diluted 1:400 in PBS in 1mL micronic tubes. One-hundred μL of the diluted samples were added to the plate which was then incubated for 45 minutes on a shaker (750rpm) at room temperature. The plate was then vacuum-washed three times with 150μL PBS per well. 100μL of a diluted secondary antibody (anti-human IgG) with a fluorescent tag (RPE) was added to each well followed by a second 45 minute incubation and vacuum wash as per previous step. Finally, 150μL PBS was added to each well and the plate placed back on a shaker at room temperature for at least 10 minutes prior to analysis.

Serum samples for the IgM immunoassay were treated with Siemens Rheumatoid factor (RF) absorbent (at a dilution of 1:2) and diluted to a final concentration of 1:800 in PBS. The plate was prepared as per the IgG immunoassay. The secondary antibody—anti-human IgM with a fluorescent RPE tag—was used in this assay for conjugation.

### Analysis

**Fig 1:**
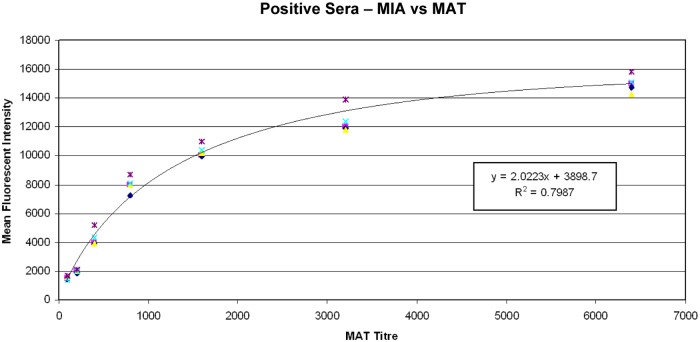
Standard curve for cut-off points.

All plate wells were then analysed using Luminex xMap technology on a BioPlex 200 Platform. The MIA results were reported as mean fluorescent intensity (MFI) and were deemed congruent or incongruent relative to the standard of comparison (MAT). Cut-off values for reactive samples were determined using five reactive sera for each MAT titre ranging from 1:100 to 1:6400 (Table 3), and developing a standard curve (R-Biopharm, 2012) using the titres obtained from MAT testing and comparing them with the mean fluorescent intensities from the MIA titrations. Fig. 1 shows the reactive sera MAT titres plotted against the MFI’s and the standard curve that resulted. From this curve, cut-off points were determined (Table 4). Positive/negative ratios were used to determine the cut-off point for non-reactive samples. During the validation and determination of cut-off points the results reactive high and reactive low were used to ensure that the MAT and the MIA results were comparable. All patient results were reported as reactive, non-reactive or equivocal.

**Table 3:** Positive sera used for standard curve.

MAT Titre	MFI				
	*Sample 1*	*Sample 2*	*Sample 3*	*Sample 4*	*Sample 5*
1:100 (5x)	1511	1422	1586	1387	1675
1:200 (5x)	2086	1879	1999	1986	2108
1:400 (5x)	4158	3897	3956	4322	5186
1:800 (5x)	7998	7258	7985	8065	8723
1:1600 (5x)	10078	9985	10203	10406	11005
1:3200 (5x)	12037	11896	11785	12403	13875
1:6400 (5x)	15106	14759	14265	15089	15843

**Table 4:** Cut-off points for reactivity equivalents of samples.

	MAT Titre	MIA IgG and IgM MFI
Non-Reactive	< 1:50	< 1200
Equivocal	1:50–1:200	1201–3999
Reactive Low	1:400–1:1600	4000–9999
Reactive High	1:3200 +	10000+

### Diagnostic Sensitivity

Sensitivity (the ability of the MIA to correctly determine the presence of leptospiral antibody) was determined by running known reactive (true positive) samples on the MIA and calculating the proportion of reactive samples detected. True positive samples are samples known to be reactive by paired sample testing with the Gold standard, the microscopic agglutination test.

### Diagnostic Specificity

Assay specificity was assessed using two methods. The first involves running known non-reactive (true negative) samples and calculating the proportion of non-reactive samples detected by the MIA. True negatives are defined as non-reactive samples known to be non-reactive by paired sample testing with the Gold standard assay, microscopic agglutination test. False positives are reactive samples determined by the test assay (MIA) that are non-reactive by the gold standard.

The second test of specificity ensured that samples that have been shown to have reactive serology for other pathogens are not cross reacting with the leptospirosis MIA.

### Assay Repeatability

Within-run repeatability was determined by running four samples 20 consecutive times on one assay run for both IgG and IgM assays. Two of these samples had an equivocal result for at least one serovar on both assays, one sample had a reactive result for at least one serovar on both assays and the remaining sample was non-reactive for all 16 serovars for both IgG and IgM assays.

Repeatability is also monitored continuously as a quality control measure by monitoring positive (reactive) and negative (non-reactive) controls with expected and accepted MFI ranges for each control serum in every assay. If the control serum results were outside of these ranges, the run was deemed to have failed and was repeated.

## Results

### MAT vs ELISA

Of the 200 samples tested, 180 samples were reactive for leptospirosis IgM by ELISA (Table 5). Twelve samples were IgM ELISA non-reactive and eight samples did not have previous IgM ELISA results; comparisons could only be made with the MAT and MIA for these eight samples. The MAT confirmed 27 of the leptospirosis IgM ELISA reactive samples had evidence of leptospiral total antibody and suggested that the remaining 153 IgM ELISA reactive samples were non-reactive (titre of < 1:50). These results suggest a substantial gap in the diagnostic performance of the ELISA and the MAT.

**Table 5:** Comparison of leptospirosis serology results for validation samples.

	MAT (Total Ab)		MIA IgG and IgM	
	REACT	NR	REACT	NR
**ELISA**				
Reactive	27 (15%)	153 (85%)	74 (41%)	106 (59%)
N = 80				
Non-Reactive	0	12	0	12
N = 12				
Not Tested	0	8	0	8
N = 8				

### MIA vs MAT

The MIA results (in mean fluorescent intensity—MFI) for the 27 MAT reactive samples also indicated reactive serology (MFI > 1200). Of the 173 non-reactive MAT samples, 126 were non-reactive on the MIA and the remaining 47 had low reactivity on the MIA, suggesting better sensitivity in the MIA. The results for five of these 47 samples, which have been confirmed as true leptospiral infections by PCR or blood culture, are shown in Table 6.

**Table 6:** MIA reactive, MAT non-reactive samples.

Sample	ELISA	MAT	MIA IgG	MIA IgM	PCR	Blood CμLt
**1**	REACTIVE	< 1:50	Equivocal	Non-Reactive	DETECTED	NEGATIVE
**2**	REACTIVE	< 1:50	Non-Reactive	Equivocal	DETECTED	POSITIVE
**3**	REACTIVE	< 1:50	Non-Reactive	Equivocal	DETECTED	POSITIVE
**4**	REACTIVE	< 1:50	Non-Reactive	REACTIVE	Not Done	POSITIVE
**5**	REACTIVE	< 1:50	Non-Reactive	Equivocal	DETECTED	Not Done

### MIA vs ELISA

The MIA detected leptospiral antibody in 74 (41%) of the 180 ELISA IgM reactive samples. The remaining 106 ELISA IgM reactive samples were non-reactive on the MIA and the non-reactive IgM ELISA samples were also non-reactive on the MIA. The 8 samples that were not previously tested by ELISA were non-reactive on the MIA also (Table 5).

### Paired Sample Testing

Of the 20 sets of additional paired samples with an MAT non-reactive acute sample and MAT reactive convalescent sample, 12 of these pairs demonstrated equivocal or reactive IgM MFI results for the acute samples with a significant rise in MFI in the convalescent samples on the IgM MIA. The results for the remaining eight pairs of samples were consistent between the MAT and the MIA. Table 7 shows the results for the paired samples comparing the MAT titre and the MIA IgM and IgG results. These samples were included in this study to show that IgM can be detected earlier or, at least at the same time, by the MIA when compared with the MAT in true leptospiral infections, as determined by a four-fold rise in serology.

**Table 7:** Comparison of MAT and MIA sensitivity in paired samples.

Sample Pair	Phase	MAT Titre	MIA IgM	MIA IgG
1	Acute	< 50	REACTIVE	Equivocal
	Convalescent	400	REACTIVE	REACTIVE
2	Acute	< 50	REACTIVE	Equivocal
	Convalescent	800	REACTIVE	REACTIVE
3	Acute	< 50	Equivocal	Equivocal
	Convalescent	800	REACTIVE	REACTIVE
4	Acute	< 50	Equivocal	Non-Reactive
	Convalescent	400	REACTIVE	REACTIVE
5	Acute	< 50	REACTIVE	Non-Reactive
	Convalescent	400	REACTIVE	Equivocal
6	Acute	< 50	REACTIVE	Equivocal
	Convalescent	1600	REACTIVE	REACTIVE
*7*	Acute	< 50	Non-Reactive	Non-Reactive
	Convalescent	800	REACTIVE	REACTIVE
8	Acute	< 50	REACTIVE	Equivocal
	Convalescent	3200	REACTIVE	REACTIVE
9	Acute	< 50	REACTIVE	Equivocal
	Convalescent	> 6400	REACTIVE	REACTIVE
*10*	Acute	< 50	Non-Reactive	Non-Reactive
	Convalescent	800	REACTIVE	Equivocal
11	Acute	< 50	Equivocal	Non-Reactive
	Convalescent	800	REACTIVE	REACTIVE
*12*	Acute	< 50	Non-Reactive	Non-Reactive
	Convalescent	400	REACTIVE	Equivocal
*13*	Acute	< 50	Non-Reactive	Non-Reactive
	Convalescent	400	REACTIVE	Equivocal
*14*	Acute	< 50	Non-Reactive	Non-Reactive
	Convalescent	400	REACTIVE	Equivocal
15	Acute	< 50	Equivocal	Equivocal
	Convalescent	1600	REACTIVE	REACTIVE
*16*	Acute	< 50	Non-Reactive	Non-Reactive
	Convalescent	400	REACTIVE	REACTIVE
17	Acute	< 50	Equivocal	Equivocal
	Convalescent	400	REACTIVE	REACTIVE
18	Acute	< 50	REACTIVE	Equivocal
	Convalescent	800	REACTIVE	REACTIVE
*19*	Acute	< 50	Non-Reactive	Non-Reactive
	Convalescent	800	REACTIVE	Equivocal
*20*	Acute	< 50	Non-Reactive	Non-Reactive
	Convalescent	400	REACTIVE	REACTIVE

### Cross-Reactivity

Of the 48 reactive viral serology samples only one showed reactive IgG and IgM serology for leptospirosis (this sample was previously reactive for Dengue virus serology) and the remaining 47 samples were non-reactive for both leptospirosis IgG and IgM.

### Repeatability

The four samples used to test within-run repeatability showed comparable results in each well across each of the 16 serovars. Table 8 shows the mean fluorescent intensity and standard deviation values for each of the four samples used in the repeatability testing for one of the serovars in the IgM immunoassay. Samples 1 and 2 were non-reactive. Sample 3 was reactive and sample 4 was in the equivocal range. The expected values were derived from comparison of the MIA mean fluorescent intensity with MAT titres. Repeatability was assessed across one run with one operator as, at the time of testing, only one operator was available to perform this testing.

**Table 8:** Assay repeatability.

Sample	Mean FI (IgM)	SD	Expected Value
1	104	4.99	< 1200
2	795	78.46	< 1200
3	4626	427.9	> 2000
4	1626	79.43	1201–2000

## Discussion

The aim of diagnostic serology is to determine reactive and non-reactive samples for a particular infectious agent. By definition, a validated assay consistently provides test results that identify samples as being reactive or non-reactive for a selected analyte, and, by inference, accurately predicts the disease status of patients with a predetermined degree of statistical certainty [21]. The aim of this study was to validate a microsphere immunoassay (MIA) using Luminex xMap technology for diagnostic leptospirosis serology screening. The validation process was performed using a comparative method—that is comparing the new assay with the current gold standard assay. Sixteen leptospiral antigens have been coupled to 16 individual magnetic bead-sets and validated as a panel for routine diagnostic leptospirosis serology. This assay gives a qualitative result—Reactive, Equivocal or Non-Reactive and has the ability to determine recent from past infection by differentiating between IgM and IgG antibodies—something that is more difficult to achieve with microscopic agglutination testing (MAT) as this test can only determine total antibody. The class of antibody detected by the MIA can be used to determine the stage of the infection which is valuable for clinicians as it can determine treatment regimens for patients or in the case of a past infection, can suggest that something other than leptospirosis is causing symptoms. Information regarding new infections is also vital from a public health perspective as it can provide information on what serovars of leptospirosis are currently circulating and indicate the areas where these infections are occurring.

All leptospirosis serology reactive samples by MAT were detected by MIA suggesting that congruence is 100% when compared to the MAT. Results from the non-reactive samples, as well as the paired samples suggest, however, that the MIA is more sensitive than the MAT. In true infections (as demonstrated by paired sample serology testing with a minimum four fold rise in titre) the MIA was able to detect low level antibody in the later stages of the acute phase as well as pick up higher levels of IgM antibody earlier in the immune phase of the infection. The MAT results indicated that these samples were non-reactive in the acute/early immune phase. The MAT generally becomes positive between day 8 and day 10 of infection [22] however, results from this validation suggest that the MIA could detect antibody in the earlier stages of infection development and increase the likelihood of the clinician submitting a convalescent sample for confirmation of infection status.

The leptospirosis IgM ELISA has previously been shown to have poor specificity, as low as 41%, when used according to the manufacturer’s instructions [22]. All leptospirosis IgM ELISA reactive samples tested in Queensland pathology laboratories are sent to the WHO/FAO/OIE Collaborating Centre for Leptospirosis Reference and Research for confirmation testing. In this study it was found that of 180 leptospirosis IgM ELISA reactive samples only 15% (27/180) of these showed reactive results on the MAT. This could be due to a lower level of antibody which is not detected by the MAT at a dilution of 1 in 50 or a non-specific antibody reaction. In this study, 41% (74/180) of the leptospirosis IgM ELISA reactive samples had reactive IgG and/or IgM serology on the MIA, again suggesting the level of antibody in these particular samples may be too low for the MAT to detect. Also, this again shows that there may be some non-specific reactions occurring in the IgM ELISA, which are not seen on the MIA. The MIA is therefore advantageous as a screening test as it reduces the large numbers of samples that are unnecessarily sent for confirmation testing by MAT. It has also been suggested that false positivity can also occur in the leptospirosis IgM ELISA due to the presence of persistent IgM from past infections [23]. The MIA screening test eliminates these results by looking at the levels of the individual IgG and IgM antibodies across paired specimens. A low level or non-reactive IgM result and a plateaued reactive IgG would be suggestive of a past infection—something not currently visible on the leptospirosis IgM ELISA or the MAT.

The MIA results suggest that the beads coated with leptospiral antigen are specific for leptospiral antibodies and show no cross-reactivity with other viral agents. The one case in this study where a Dengue Virus reactive serology sample also showed leptospiral antibodies is likely to be a true leptospirosis infection occurring simultaneously with a Dengue Virus infection. Leptospirosis and Dengue Virus infections are both common in northern parts of Queensland (where this sample was from) as they are both associated with tropical and sub-tropical regions where extreme weather events occur [24]. In many cases samples are submitted for both arbovirus testing (including Dengue virus) and leptospirosis testing at the same time.

The results from the MIA show that reproducibility is possible and accurate when compared to the MAT. A major disadvantage of the MAT is attenuation of the live leptospiral cultures. It has been shown that over time, leptospiral cultures lose their antigenicity and therefore become less effective [25]. Also, day to day, the cultures can be different—more or less dense or contaminated—which makes reproducing results accurately a difficult task on the MAT. This issue is overcome with the MIA as the antigens (leptospiral cultures) are wild type cultures with a known passage number and are all diluted to a known concentration (1.8 x 10^9^) prior to the bead coupling process. This ensures that there are equal amounts of each antigen available in every test. Another major advantage of the MIA over the MAT is that there is no need to maintain stocks of live leptospiral cultures for daily use. Pure cultures are only used in the MIA as antigens for bead coupling and these antigens can be centrifuged, diluted and frozen at -20°C for up to six months [26]. Currently, performing the MAT on a routine basis requires sub-culturing more than 200 tubes per week, maintaining four stocks of cultures.

When comparing the MAT and the MIA the advantages of the latter are obvious. Firstly, the MIA is less time consuming—a full plate of 88 samples can be run in around three hours. To run the same number of samples on the MAT, it would take twice the time for a full panel of 16 serovars excluding analysis. The MIA is also less labour intensive as it does not require adding 16 individual cultures to each well on a 96 well plate for each individual patient. These savings combined as well as the reagent costs suggest that the MIA is also less costly than the MAT. An analysis of laboratory and assay costs shows that the current diagnostic serology method (MAT) is performed at a cost of $AUD6.95 (excluding labour) to the leptospirosis reference laboratory per sample per 16 serovars [27]. In comparison, the MIA costs $AUD4.95 per sample (excluding labour) per 16 serovars. Secondly, the MIA uses a total of 7μL of serum (2μL for the IgG assay dilutions and 5μL for the IgM assay dilutions) compared with 50μL of serum used in the MAT. Thirdly, the MIA has the ability to detect and differentiate both IgG and IgM antibodies whereas the MAT can only detect total antibody and cannot give an accurate indication of the stage of infection in a single sample. The MIA can potentially include up to 500 analytes in the one assay, therefore, there is potential to be able to include all known leptospirosis serovars (~250) in one test at one time. Given the number of bead-sets available for microsphere immunoassays other applications could potentially involve the inclusion of a number of different viral and bacterial agents in one assay. For example, leptospirosis antibody detection and Dengue Virus antibody detection could be combined into one routine diagnostic test.

In conclusion, the results from this validation suggest that the leptospirosis MIA is a beneficial diagnostic screening tool for leptospirosis serology testing. This assay is able to determine reactive, equivocal and non-reactive samples when compared to the MAT. It is able to differentiate leptospiral IgG antibodies from leptospiral IgM antibodies which will provide vital diagnostic information as well as provide a better epidemiological picture. Further investigations will include validation of each individual serovar to enable serovar specific results to be reported and validation of a microsphere immunoassay for detection of leptospiral antibodies in animal samples will also be looked at in the future.

## Supporting Information

S1 Flowchart (DOC)

S1 Checklist STARD Checklist. (DOC)
