# Supplementary material for: Validation of a Microsphere Immunoassay for Serological Leptospirosis Diagnosis in Human Serum by Comparison to the Current Gold Standard
Source: PLoS Negl Trop Dis. 2015 Mar 25;9(3):e0003636. doi: 10.1371/journal.pntd.0003636 (PMC4373873; doi:10.1371/journal.pntd.0003636)
Supplement: Supplementary file 2 [file pntd.0003636.s001.doc]

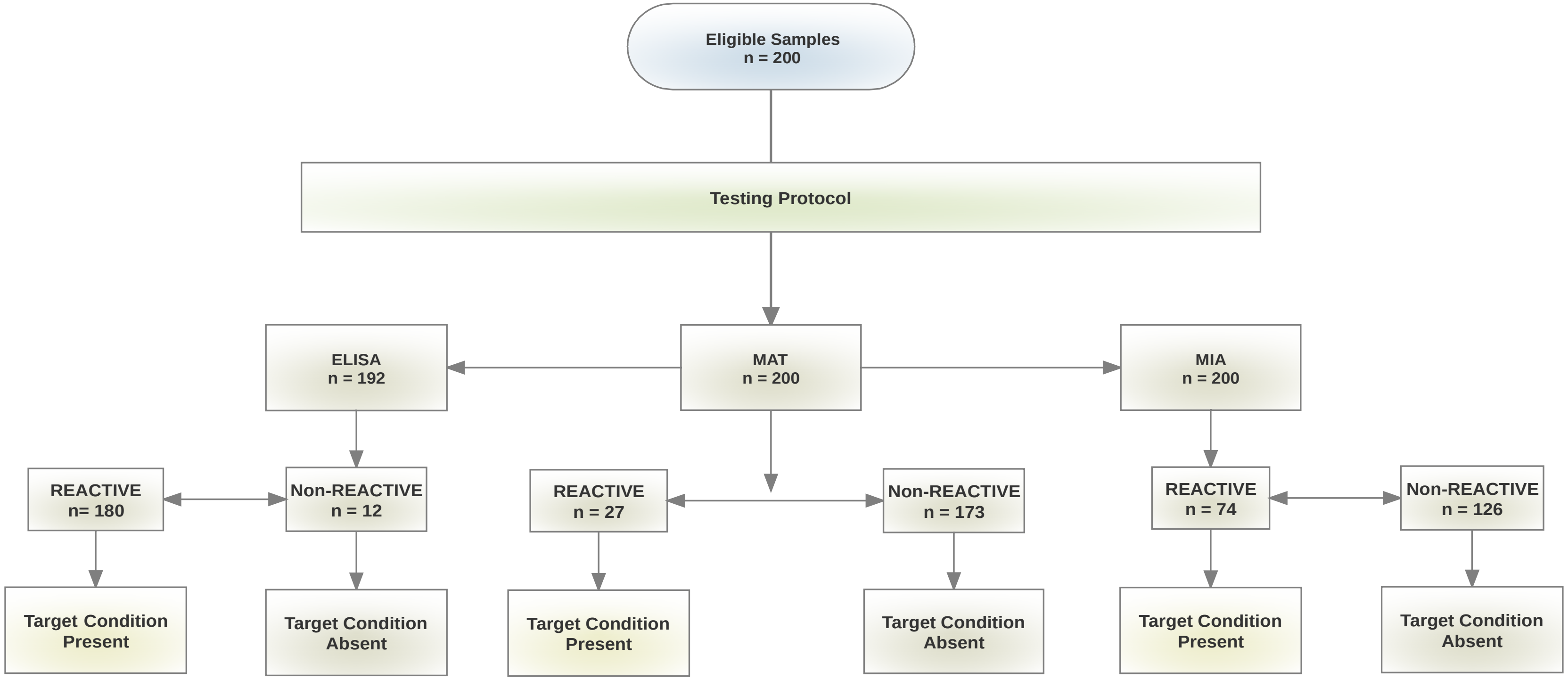
Eligible Samples
n = 200
Testing Protocol
ELISA
n = 192
MAT
n = 200
MIA
n = 200
REACTIVE
n= 180
Non-REACTIVE
n = 12
REACTIVE
n = 27
Non-REACTIVE
n = 173
REACTIVE
n = 74
Non-REACTIVE
n = 126
Target Condition Present
Target Condition Absent
Target Condition Present
Target Condition Absent
Target Condition Present
Target Condition Absent
